# *Apocynum venetum* L. leaves extract inhibits ferroptosis and improves renal fibrosis in diabetic kidney disease by regulating the Nrf2/HO-1 pathway

**DOI:** 10.3389/fphar.2026.1807373

**Published:** 2026-05-07

**Authors:** Guohua Shi, Subinuer Abuduaini, Li Chen, Subinuer Erreken, Mei Long, Xiaoqian Tang, Jinsen Kang

**Affiliations:** 1 College of Pharmacy, Xinjiang Medical University, Urumqi, China; 2 State Key Laboratory Basis of Xinjiang Indigenous Medicinal Plants Resource Utilization and The Key Laboratory of Plant Resources and Chemistry of Arid Zone, Xinjiang Technical Institute of Physics and Chemistry, Chinese Academy of Sciences, Urumqi, China; 3 Department of Pharmacology, School of Pharmacy, Xinjiang Medical University, Xinjiang Key Laboratory of Natural Medicines Active Components and Drug Release Technology, Xinjiang Key Laboratory of Biopharmaceuticals and Medical Devices, Engineering Research Center of Xinjiang and Central Asian Medicine Resources, Ministry of Education, Urumqi, China

**Keywords:** diabetic kidney disease, ferroptosis, Apocynum venetum, Nrf2, HO-1

## Abstract

**Background:**

Diabetic kidney disease (DKD) is a common microvascular complication of diabetes. Current treatments for DKD are limited and associated with side effects. *Apocynum venetum* L. leaves, a traditional herbal medicine, have pharmacological potential for DKD but their mechanisms of action remain unclear.

**Purpose:**

Investigating the Pharmacological Effects and Mechanisms of *Apocynum venetum* L. leaves against DKD.

**Methods:**

Using db/db mice as an *in vivo* model, *Apocynum venetum* L. leaves extract (AVLE) was administered via oral gavage for 12 weeks. Serum and urine parameters related to renal function were measured, and renal tissue histopathology staining was performed. *In vitro*, human renal tubular epithelial cells (HK-2) stimulated with high glucose (HG) were used to evaluate fibrosis-related protein expression and the pharmacological effects of AVLE. UHPLC-OE-MS was used to analyze the metabolites absorbed into serum after AVLE administration, proteomic analysis was employed to explore the potential mechanisms, and the key signaling pathways and protein expression levels were verified *in vitro* and *in vivo*. Proteomic analysis suggested that ferroptosis may be a key pathway, and HO-1 may be a potential target.

**Results:**

Treatment with AVLE significantly reduced proteinuria, renal pathological damage, and fibrosis in db/db mice and mitigated HG-induced injury in HK-2 cells. A total of 60 metabolites absorbed into the serum of AVLE were identified. Proteomic analysis suggests that ferroptosis may be a key pathway, and HO-1 may be a potential target. Subsequent experimental verification showed that AVLE inhibited ferroptosis and regulated the Nrf2/HO-1 signaling pathway.

**Conclusion:**

AVLE can improve diabetic kidney disease and is closely associated with the regulation of the Nrf2/HO-1 signaling pathway, thereby alleviating renal dysfunction, fibrosis, and oxidative stress. This study provides experimental evidence for the potential application of AVLE in the prevention and treatment of diabetic kidney disease.

## 1 Introduction

According to the 11th edition of the IDF Diabetes Atlas, the global population of individuals aged 20 to 79 with diabetes reached 589 million in 2024, with a prevalence rate of 11.1%, imposing a significant burden on both health and the economy (Duncan et al., 2025). Managing diabetes and its associated complications, including cardiovascular disease, renal failure, and retinopathy, continues to present a substantial and ongoing challenge (Kalyani et al., 2025). The most frequent cause of end-stage renal disease (ESRD) is DKD, a dangerous side effect of diabetes (Umanath and Lewis, 2018). Its clinical features include progressive decline in renal function, with or without proteinuria. The progression and development of DKD are influenced by various factors, including oxidative stress, inflammation, apoptosis, lipid accumulation, fibrosis, and hyperglycemia (Ambinathan et al., 2021; Ricciardi and Gnudi, 2021; Jung and Yoo, 2022). Current strategies to treat DKD include glycemic control and blood pressure management (Selby and Taal, 2020). However, they fail to effectively halt the progression of DKD, necessitating the urgent search for suitable treatment methods.

**TABLE 1:** Antibody information used in Western blotting analysis.

Antibody	Dilution	Manufacturer	Cat.no
Nrf2 rabbit polyclonal antibody	1:1200	Affinity	AF0639
GPX4 rabbit monoclonal antibody	1:5000	HUABIO	ET1706-45
KIM-1 rabbit monoclonal antibody	1:1000	HUABIO	HA721535
SLC7A11 rabbit monoclonal antibody	1:1000	HUABIO	HA721868
HO-1 rabbit polyclonal antibody	1:3000	Proteintech	10701-1-AP
α-SMA rabbit polyclonal antibody	1:7000	Proteintech	14395-1-AP
E-cadherin rabbit polyclonal antibody	1:100000	Proteintech	20874-1-AP
Fibronectin rabbit polyclonal antibody	1:11000	Proteintech	15613-1-AP
Beta actin rabbit polyclonal antibody	1:10000	Proteintech	81115-1-RR
HRP-goat anti-rabbit recombinant secondary antibody	1:10000	Proteintech	RGAR001

An iron-dependent form of programmed cell death known as ferroptosis is typified by iron overload, which results in mitochondrial malfunction and the buildup of reactive oxygen species (ROS) (Rochette et al., 2023) and elevated lipid peroxidation (Chen et al., 2024). Ferroptosis has been linked to a range of conditions, such as cancer (Jiang et al., 2021), aging, neurological disorders (Ma et al., 2022), cardiovascular disease (Zhao et al., 2025), and kidney disease. Inhibiting ferroptosis may be a treatment option for DKD, as ferroptosis contributes to its development. In recent years, research has shown that activation of the Nrf2/HO-1 pathway suppresses ferroptosis in DKD by reducing Fe^2+^ accumulation, lipid peroxidation, and mitochondrial damage (Wang et al., 2024). Several natural products, including quercetin (Feng et al., 2023) and grape seed proanthocyanidin extract (Li et al., 2025), exert renoprotective effects in DKD by targeting this pathway to inhibit ferroptosis. Therefore, we hypothesized that AVLE exerts renoprotective effects by regulating the Nrf2/HO-1 pathway to inhibit ferroptosis.

Natural products demonstrate significant potential in the prevention and treatment of DKD (Chen et al., 2022). Dried leaves of *Apocynum venetum* L., which belongs to the family Apocynaceae, are known as *A. venetum* L. Their medicinal use has been documented since before the Han Dynasty (Xie et al., 2025). The blood pressure-lowering effects of *A. venetum* L. have been reported domestically and internationally (Lau et al., 2012). *Apocynum venetum* L. leaves also exert antidepressant (Li et al., 2018), antioxidant, anti-inflammatory (Liu et al., 2022), and blood lipid regulatory effects. The main components of *Apocynum* include flavonoids, catechins, organic acids, fatty acid esters, alcohols, sterols, and sugars (Jiang et al., 2025). Modern treatment strategies for diabetic kidney disease have shifted from solely controlling blood glucose to intervening in oxidative stress and chronic inflammation (Efiong et al., 2025). The flavonoids and other components rich in *A. venetum* L. leaves have antioxidant and anti-inflammatory properties (Xie et al., 2025). AVLE reportedly improves impaired renal function in diabetic rats (Chen et al., 2010). However, the material basis and specific mechanisms of action remain unclear.

Building on our previously established extraction protocol for AVLE (Si et al., 2025), we evaluate the protective effects of AVLE on DKD through *in vitro* and *in vivo* experiments, and identify the metabolites absorbed into serum of AVLE using UHPLC-OE-MS technology. Subsequently, proteomic analysis is performed on kidney tissues after AVLE intervention to explore its potential mechanisms of action, which are then verified by experiments.

## 2 Methods

### 2.1 Materials and methods

The dried leaves of *Apocynum venetum* L. were collected from the GABAU KENDER Resource Reserve (Altay, Xinjiang, China) and authenticated by Dr. Chunfang Lu at the Xinjiang Technical Institute of Physics and Chemistry, Chinese Academy of Sciences. HK-2 cells (GDC0152) were purchased from the China Center for Type Culture Collection (CCTCC, China). Metformin hydrochloride (Solarbio, 24240726009) was obtained from Solarbio. Tissue fixative (Biosharp Technology Co., Ltd., BL539A) was purchased from Biosharp Technology Co., Ltd. D-(+)-Glucose (Sigma, G7021-100G) and D-mannitol (Biofroxx, EZ66EAB7D2) were purchased from Sigma and Biofroxx, respectively. Zinc protoporphyrin (ZnPP) (MedChemExpress, HY-101193) was purchased from MedChemExpress. Dulbecco’s Modified Eagle Medium/Nutrient Mixture F-12 (DMEM/F12) sugar-free medium (Wuhan Punoise Life Science Co., Ltd., WH4224D58) was obtained from Wuhan Punoise Life Science Co., Ltd. The CCK-8 assay kit (Elabscience, WU05F8R04824) and BCA Protein Concentration Kit (Solarbio, 240012029) were purchased from Elabscience and Solarbio, respectively. Urine albumin (mALB) kits were purchased from Elabscience (E-EL-M0792). MDA, SOD, GSH, and LDH kits were purchased from Nanjing Jiancheng Bioengineering Institute (20250409; 20250407; 20250409; 20250412, Jiangsu). ROS kits were purchased from Elabscience (E-BC-K138-F). IL-6, IL-1β, and TNF-α kits were purchased from FineTest (ER1393, ER0042, ER1393).

### 2.2 Preparation of AVLE

*Apocynum venetum* L. leaves (250 g) were pulverized using a grinder in several small batches. The powdered material was then extracted twice by continuous reflux with 50% (v/v) ethanol (7,500 mL each) for 1 h per extraction (Si et al., 2025). The combined extract was further purified using HPD300 macroporous resin. The column was initially rinsed with three bed volumes of 50% ethanol to clear out any unwanted contaminants, before subsequently being subjected to elution with one bed volume of 70% ethanol. The resulting eluate was then collected, concentrated, and preserved through freeze-drying techniques. The extraction yield was 8.97%.

### 2.3 Animals and cells

#### 2.3.1 Animal experiments

Six-week-old db/db (C57BLKS/J) mice and age-matched db/m control mice were purchased from Changzhou Cavens Biological Co., Ltd. and housed in the SPF animal facility at the Animal Experiment Center of Xinjiang Medical University. The animals were maintained under standard 12 h light/12 h dark cycles, a temperature of 22 °C ± 2 °C, and a humidity of 40%–60%. Adequate water and food were provided throughout the experiment. The animals were acclimated for 2 weeks before the trial. Xinjiang Medical University’s Animal Care and Ethics Committee approved all animal experiments (Approval No.: IACUC-JT-20240226-55).

According to the recommendations of the Chinese Pharmacopoeia, the daily human dose of *A. venetum* L. leaves is 6–12 g. This study uses a daily human dose of 12 g as the basis for dose conversion. Using the calculation method, this was converted to the equivalent mouse dose, and based on this, the low, medium, and high dose groups in the AVLE study were set at 140, 280, and 560 mg/kg/day, respectively. Based on their body weight and blood glucose levels, the db/db mice were randomly divided into five groups, with six animals in each group. Each group was treated as follows: MOD group (db/db); AVLE-L group (db/db + 140 mg/kg AVLE); AVLE-M group (db/db + 280 mg/kg AVLE); AVLE-H group (db/db + 560 mg/kg AVLE); and Metformin (MET)group (db/db + 200 mg/kg MET) (Saberi-Hasanabadi et al., 2025). An additional 6 db/m mice served as the CON group (db/m). After 12 weeks of intervention, mouse body weight and blood glucose were measured, with 24-h urine, blood, and kidney tissue collected for subsequent analysis.

#### 2.3.2 Biochemical analysis

A biochemical analyzer was used to test the levels of Serum Creatinine (SCr) and Blood Urea Nitrogen (BUN) in mouse serum (Leidu Life Science, Shenzhen, China). Cystatin C (Cys-C) was determined using a commercial assay kit. Twenty-four-hour urine collection (24 h-UTP) was performed to measure urine albumin (mALB) and urinary creatinine (Ucr) using a specific assay kit, and the Urine Albumin-to-Creatinine Ratio (uACR) was calculated accordingly.

#### 2.3.3 Histopathological staining

After kidney tissues were fixed in 4% paraformaldehyde, they were sectioned, embedded in paraffin, and stained with HE, Masson, and PAS.

#### 2.3.4 Transmission electron microscopy (TEM)

Mouse kidney tissue was fixed, dehydrated, and embedded in 812 epoxy resin. Ultrathin sections (60-80 nm) were cut, stained with lead citrate and uranyl acetate, and subsequently examined under a transmission electron microscope (HITACHI HT7700, operating at 80 kV).

#### 2.3.5 Immunohistochemistry

After dewaxing, kidney tissue sections were subjected to antigen retrieval by heating in sodium citrate buffer at 100 °C. Following this procedure, the sections underwent blocking with a 5% solution of bovine serum albumin for 1 hour, were thereafter exposed to primary antibodies and incubated overnight at 4 °C, and finally, received secondary antibodies with an additional 1-h incubation period. Signals were detected using DAB. The sections were imaged and analyzed using electron microscopy, and positive areas were quantified using ImageJ software.

#### 2.3.6 Cell culture and grouping

HK-2 cells were maintained in DMEM/F12 medium enriched with 1% penicillin/streptomycin and 10% fetal bovine serum, incubated at 37 °C in an atmosphere containing 5% CO_2_. The cells were then divided into normal-glucose (NG, 5.5 mM glucose), high-mannitol (HM, 5.5 mM glucose +54.5 mM mannitol), and high-glucose (HG, 60 mM glucose) groups, AVLE-L group (HG + 12.5 μg/mL AVLE), AVLE-M group (HG + 25 μg/mL AVLE), AVLE-H group (HG + 50 μg/mL AVLE), and ZnPP group (HG + 1 µM ZnPP). ZnPP is an HO-1 inhibitor.

#### 2.3.7 Cell viability assay

HK-2 cells (5,000 cells/well) were seeded into 96-well plates. After 24 h of culture, the cells were treated with different concentrations of AVLE or ZnPP for 48 h and then incubated with Cell Counting Kit-8 reagent at 37 °C for 2 h. An enzyme-linked immunosorbent assay reader was used to quantify the absorbance at 450 nm in each well (Tecan Austria GmbH, INFINITE E PLEX, Austria).

#### 2.3.8 ROS generation detection

HK-2 cells were treated accordingly and incubated for 30 min at 37 °C in the dark with serum-free DMEM/F12 medium containing 10 μM carboxy-H2DCFDA. After washing with PBS, images were acquired using a fluorescence microscope (DMi8, Leica, Germany).

#### 2.3.9 Ferrous iron level assay

The amount of ferrous iron in kidney tissue and HK-2 cells was measured using a commercial kit. In brief, 30 mg of kidney tissue was ground using a tissue grinder (Seville Company, Wuhan, China). Then, 1 × 10^6^ cells grown in T25 flasks were collected and sonicated for homogenization. Following the kit instructions, the supernatant was gathered for further analysis.

#### 2.3.10 Oxidative stress level detection

Detect MDA, GSH, and SOD levels in kidney tissue and HK-2 cells, respectively. Follow the kit instructions and measure the content of each sample using an enzyme-linked immunosorbent assay reader at the corresponding wavelength. Standardize by total protein content in the kidney homogenate or cell lysate.

#### 2.3.11 Enzyme-linked immunosorbent assay

Using the appropriate ELISA kits, the levels of IL-1β, TNF-α, and IL-6 in kidney tissue and HK-2 cells were determined.

#### 2.3.12 Western blot analysis

Kidney tissue and HK-2 cells were lysed using RIPA lysis solution, including protease inhibitors. After that, the total protein was isolated and quantified. Proteins were transferred onto PVDF membranes after being separated via SDS-PAGE and blocked with 5% nonfat milk. After that, they were incubated with primary antibodies for the entire night (KIM-1, α-SMA, E-cadherin, FN, GPX4, SLC7A11, Nrf2, HO-1, and β-actin; see Table 1 for details) at 4 °C. After three 10-min TBST washes, the samples were incubated with secondary antibodies for 1 hour. Using an ECL kit, protein bands were found, and ImageJ software was used to quantify their intensity.

### 2.4 Preparation of AVLE-containing serum

Six-week-old Sprague–Dawley rats were purchased from the Animal Experiment Center of Xinjiang Medical University. After a week of acclimatization, the rats were administered 1 g/kg/day AVLE (equivalent to 10 times the adult human dose when adjusted for body surface area). The control group received an equal volume of saline. Gastric lavage was performed once daily for seven consecutive days. Serum was collected 2 h after the final gavage (Yan et al., 2024).

### 2.5 UHPLC-OE-MS analysis

Separation was conducted on a Vanquish ultra-high performance liquid chromatograph (Thermo Fisher Scientific) coupled with a Phenomenex Kinetex C18 column (2.1 mm × 100 mm, 2.6 μm). The mobile phase system comprised an aqueous solution with 0.01% acetic acid as solvent A and an isopropanol-acetonitrile mixture (1:1, v/v) as solvent B. Sample tray temperature was controlled at 4 °C, with an injection volume of 2 μL. Mass spectrometric scanning was performed on an Orbitrap Exploris 120 instrument operated via Xcalibur software (version 4.4). Both full-scan and tandem mass spectra were recorded under optimized parameters: sheath gas flow set to 50 Arb, auxiliary gas flow at 15 Arb, capillary temperature maintained at 320 °C, primary MS resolution of 60,000, MS/MS resolution of 15,000, stepped normalized collision energy at 20/30/40, and ionization voltage of 3.8 kV in positive mode or −3.4 kV in negative mode.

### 2.6 Proteomics research

Mouse kidney samples were extracted via freezing, homogenization, and centrifugation, followed by protein concentration determination using the BCA assay and assessment of sample quality via SDS-PAGE and Coomassie Blue staining. Protein samples that passed quality control underwent trypsin digestion for peptide quantification. Equal quantities were processed using single-run Data-Independent Acquisition (DIA). The differentially expressed proteins identified by proteomics were uploaded to the Metascape online platform for GO enrichment analysis and KEGG pathway analysis. The entries were sorted into biological process (BP), cellular component (CC), molecular function (MF), and KEGG pathways, followed by visualization of the results.

### 2.7 Statistical analysis

All statistical analyses were performed using Prism 9.5.1 software (GraphPad, La Jolla, CA, USA). Data from two or more groups were analyzed using one-way ANOVA, with *p* < 0.05 considered statistically significant.

## 3 Results

### 3.1 Effects of AVLE on renal injury in db/db mice

**FIGURE 1:**
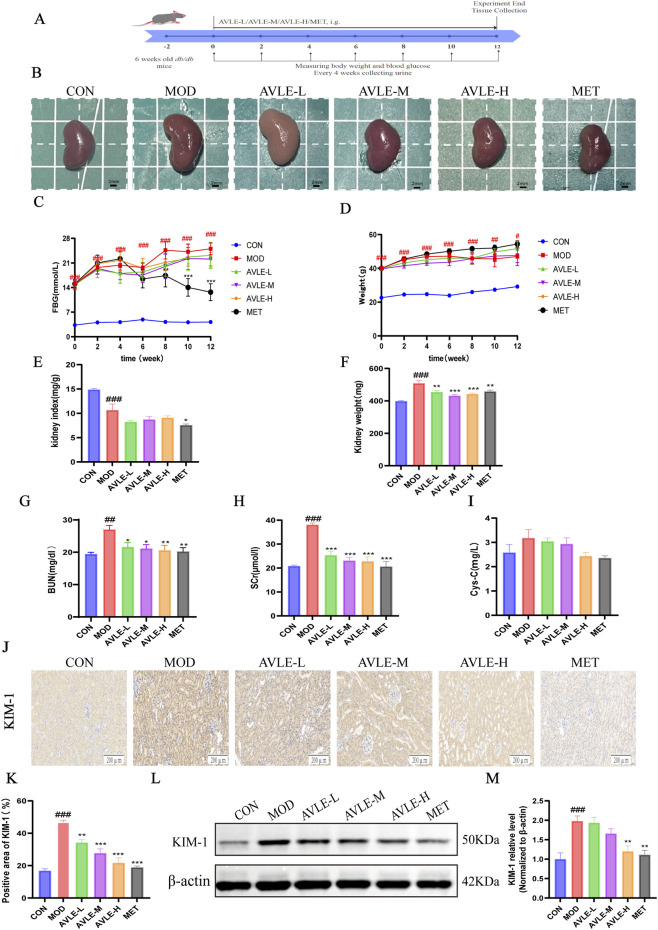
The effect of AVLE on the kidneys of db/db mice **(A)** Experimental design **(B)** Changes in kidney volume across groups **(C,D)** Blood glucose levels and body weight were measured at different time points **(E,F)** Changes in kidney weight index across groups **(G-I)** Serum BUN, SCr, and Cys-C levels in each group **(J)** The expression of KIM-1 protein was detected by the IHC method. Scale bar = 200 μm **(K)** Semi-quantitative analysis of KIM-1 protein immunohistochemistry **(L,M)** KIM-1 protein expression in renal lysates, with β-actin as the internal control. Data are expressed as mean ± SEM (n = 6). Compared with the CON group: ^##^ *p* < 0.01, ^###^ *p* < 0.001; compared with the MOD group: ^*^ *p* < 0.05, ^**^ *p* < 0.01, ^***^ *p* < 0.001.

Using db/db mice as an *in vivo* model (Figure 1A), the MOD group exhibited significant increases in kidney volume, Fasting Blood Glucose (FBG), body weight, kidney weight, and kidney index after 12 weeks. Treatment with different doses of AVLE significantly improved renal hypertrophy in db/db mice, reducing kidney weight and index. However, AVLE treatment exerted minimal effects on blood glucose and body weight (Figures 1B–F).

To further evaluate the protective effect of AVLE on renal function in db/db mice, we measured BUN and SCr levels. Compared with the CON group, BUN and SCr levels were elevated in the MOD group (*p* < 0.01). Following treatment with different doses of AVLE, renal function indicators in db/db mice improved, manifested as a dose-dependent decrease in BUN and SCr levels (*p* < 0.05). The ameliorative effect of high-dose AVLE was comparable to that of the positive control drug, metformin (Figures 1G,H). Cys-C levels showed a decreasing trend following AVLE treatment, although the difference did not reach statistical significance (Figure 1I). Tubular cells take up fatty acids *via* KIM-1, thereby inducing tubular injury accompanied by interstitial inflammation and diabetic kidney disease fibrosis (Mori et al., 2021). IHC and Western blot analyses of kidney tissue revealed elevated KIM-1 protein expression in the MOD group (*p* < 0.001), which decreased following AVLE treatment (Figures 1J–M). These findings indicate that AVLE exerts a protective effect on renal function in db/db mice.

### 3.2 Impact of AVLE on renal pathological damage and urinary protein excretion in db/db mice

**FIGURE 2:**
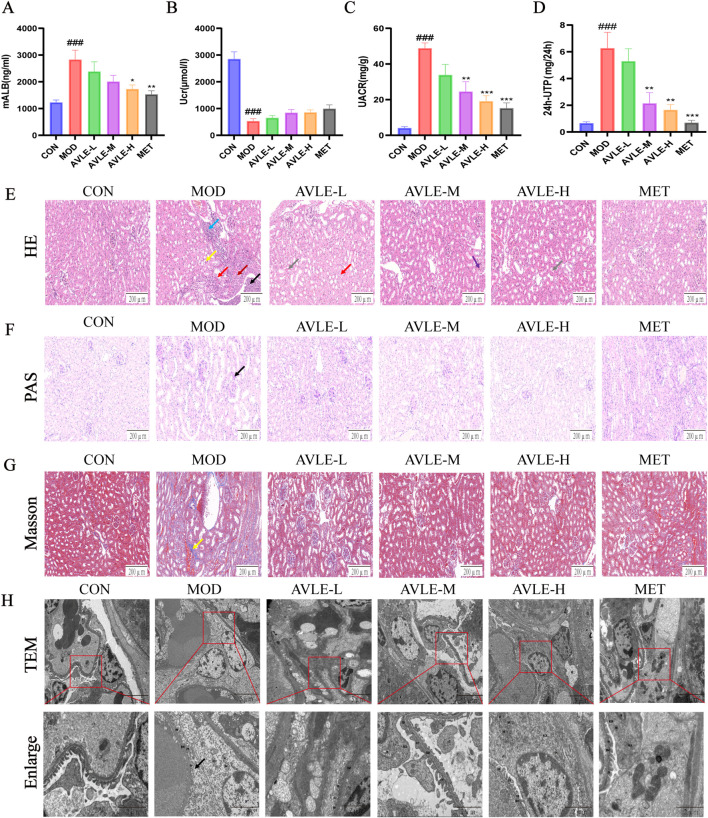
Impact of AVLE on renal pathological deterioration and urine protein excretion in db/db mice. **(A–D)** Measurement of mALB, Ucr, uACR, and 24 h-UP in mouse urine. **(E–G)** Renal tissue HE, Periodic Acid-Schiff (PAS), and Masson. Scale bar = 200 μm. **(H)** Representative TEM images of renal lesions in db/db mice. Scale bar = 5 or 2 μm. All data are expressed as mean ± SEM (n = 6). Compared with the CON group, ^###^ *p* < 0.001; compared with the MOD group, ^*^ *p* < 0.05, ^**^ *p* < 0.01, ^***^ *p* < 0.001.

The MOD group had significantly higher levels of mALB, uACR, and 24 h-UP than the CON did, suggesting considerable proteinuria (*p* < 0.01). The level of proteinuria decreased after AVLE and MET were administered (Figures 2A–D).

Renal histopathology revealed tubular epithelial cell edema in the MOD group, accompanied by fibroblast proliferation and inflammatory cell infiltration. Necrotic cell debris was observed (Figure 2E), along with glycogen deposition and thickening of the glomerular mesangial matrix (Figure 2F). Collagen fiber proliferation was visible peritubularly, appearing blue or bright blue with irregular shapes and disordered arrangement (Figure 2G). The AVLE and MET groups showed significant improvement. TEM images revealed extensive cytoplasmic dissolution in the podocytes of MOD mice, along with swollen organelles and widespread fusion of foot processes. By contrast, the AVLE and MET groups exhibited relatively milder damage with intact podocyte membranes, abundant cytoplasm, and reduced foot process fusion (Figure 2H). These findings indicate that AVLE ameliorates renal histopathological damage in db/db mice.

### 3.3 Impact of AVLE on renal fibrosis-related proteins in db/db mice

**FIGURE 3:**
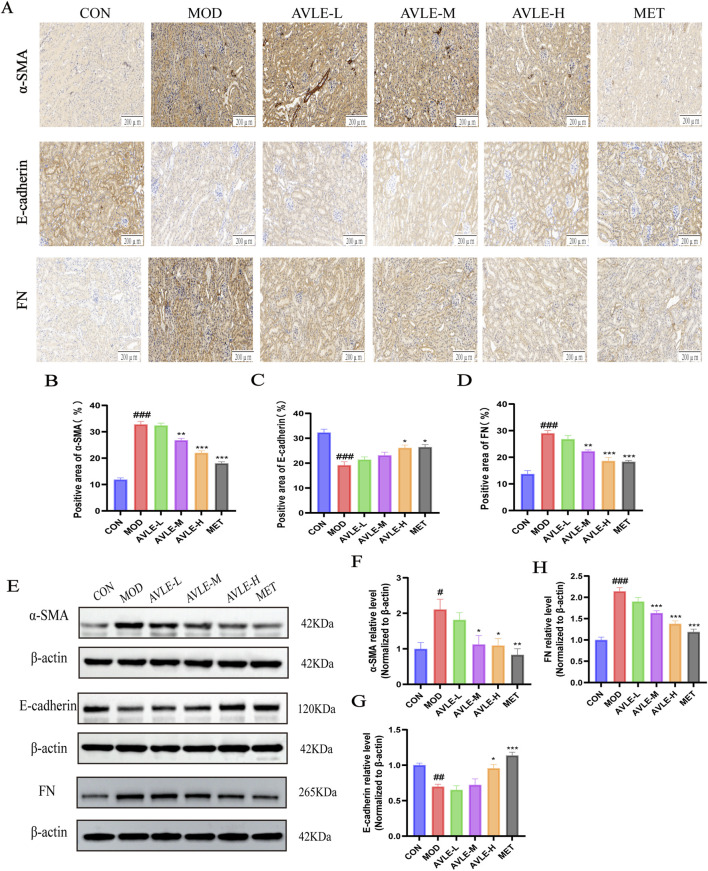
Effects of AVLE on fibrosis-related proteins in db/db mouse kidneys. **(A–D)** Immunohistochemical detection and semi-quantitative analysis of α-SMA, E-cadherin, and FN expression in kidney tissue. Scale bar = 200 μm. **(E–H)** Expression of α-SMA, E-cadherin, and FN in kidney lysates, with β-actin as the internal control. All data are expressed as mean ± SEM (n = 6). Compared with the CON group: ^#^ *p* < 0.05, ^##^ *p* < 0.01, ^###^ *p* < 0.001; compared with the MOD group: ^*^ *p* < 0.05, ^**^ *p* < 0.01, ^***^ *p* < 0.001.

To investigate whether AVLE exerts renal protective effects by modulating the fibrotic process, immunohistochemistry and Western blot analyses were performed to detect the expression of epithelial-mesenchymal transition and fibrosis-related markers in renal tissues. Compared with the CON group, the MOD group exhibited significantly reduced E-cadherin expression (*p* < 0.01), while α-SMA and FN expression were significantly upregulated (*p* < 0.05). Compared with the MOD group, both the AVLE-H and MET groups significantly increased E-cadherin expression (Figures 3A–H). AVLE-M, AVLE-H, and MET groups all significantly downregulated α-SMA and FN protein expression levels. These results indicate that AVLE possesses anti-fibrotic properties (*p* < 0.01).

### 3.4 Impact of AVLE on oxidative stress and inflammatory mediators in db/db mice

**FIGURE 4:**
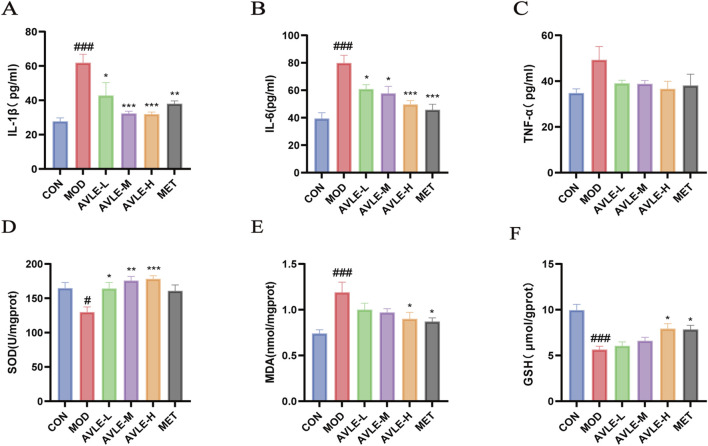
AVLE’s effects on oxidative stress and inflammatory variables in db/db mice. **(A–F)** IL-1β, IL-6, TNF-α, SOD, MDA, and GSH levels in mouse kidneys were measured using kits. All data are expressed as mean ± SEM (n = 6). Compared with the CON group: ^#^ *p* < 0.05, ^###^ *p* < 0.001; Compared with the MOD group: ^*^ *p <* 0.05, ^**^ *p <* 0.01, ^***^ *p <* 0.001.

To further assess the renoprotective effects of AVLE, the levels of inflammatory cytokines and oxidative stress markers in renal tissue homogenates were measured in db/db mice. The MOD group showed higher IL-1β, IL-6, and TNF-α (*p* < 0.001), indicating severe inflammation. AVLE and MET exhibited significantly reduced IL-1β and IL-6 levels and decreasing TNF-α. These results indicate that AVLE improves renal inflammatory responses in db/db mice (Figures 4A–C). The MOD showed lower SOD activity and GSH and markedly higher MDA content (*p* < 0.05). Treatment with AVLE and MET reduced oxidative stress levels (Figures 4D–F). These results indicate that AVLE reduces oxidative stress in the kidneys of db/db mice.

### 3.5 Impact of AVLE on inflammation, oxidative stress, and damage caused by high glucose in HK-2 cells

**FIGURE 5:**
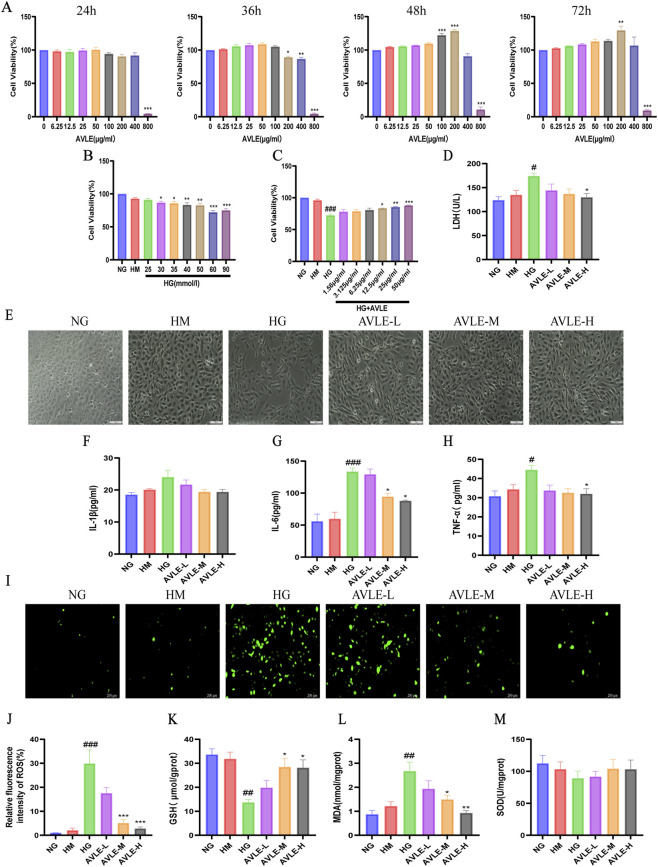
Impact of AVLE on oxidative stress, inflammation, and damage caused by elevated hyperglycemia in HK-2 cells. **(A)** Cell viability of normal HK-2 cells treated with varying concentrations of AVLE, compared with the 0 μg/mL: *p < 0.05, **p < 0.01, ***p < 0.001. **(B)** Effect of HG on HK-2 cell viability. **(C–H)** Effects of AVLE on high-glucose-induced HK-2 cells: **(C)** cell viability, **(D)** LDH release, **(E)** cell morphology, **(F)** IL-1β levels, **(G)** IL-6 levels, and **(H)** TNF-α levels. **(I)** Representative fluorescence images of carboxy-H2DCFDA-stained HK-2 cells. Scale bar = 200 μm. **(J)** ROS levels in HK-2 cells (K–M) Effects of AVLE on **(K)** GSH levels **(L)** MDA levels, and **(M)** SOD activity in high-glucose-treated HK-2 cells. All data are expressed as mean ± SEM (n = 3). Compared with the NG group: ^#^ *p* < 0.05, ^##^ *p* < 0.01, ^###^ *p* < 0.001; compared with the HG group: ^*^ *p* < 0.05, ^**^ *p* < 0.01, ^***^ *p* < 0.001.

AVLE did not exhibit significant toxic effects within the concentration range of 0–50 μg/mL (Figure 5A). A HG-stimulated HK-2 cell model was established (Figure 5B) (Zhang et al., 2020). To examine the impact of AVLE on damage to renal tubular cells under hyperglycemic circumstances, HK-2 cells were exposed to 60 mM of high glucose and subsequently treated with varying dosages of AVLE. Following a 48-h period, the viability of cells in the HG group was markedly reduced compared to the normal NG group (*p* < 0.001). Treatment with AVLE at concentrations of 12.5, 25, and 50 μg/mL notably enhanced cell viability in HG conditions in a dose-dependent manner (Figure 5C). No significant difference in LDH release rates was seen between the CON and HM groups, showing that osmotic pressure was not the key driver. The HG group exhibited significantly elevated LDH release. After 48 h, cell viability in the HG group decreased significantly, while AVLE treatment resulted in a marked improvement (Figure 5D). Following HG stimulation, HK-2 cell morphology shifted from cobblestone-like to elongated spindle cells, demonstrating marked morphologic alteration. AVLE intervention restored cellular morphology (Figure 5E). The HG group exhibited elevated IL-6, TNF-α, and IL-1β (*p* < 0.05), which were partially suppressed by AVLE intervention (Figures 5F–H). The HG group demonstrated increased ROS production, elevated MDA, and decreased GSH and SOD activity (*p* < 0.05). AVLE intervention markedly improved these parameters (Figures 5I–M).

### 3.6 Identification of AVLE metabolites absorbed into serum

**FIGURE 6:**
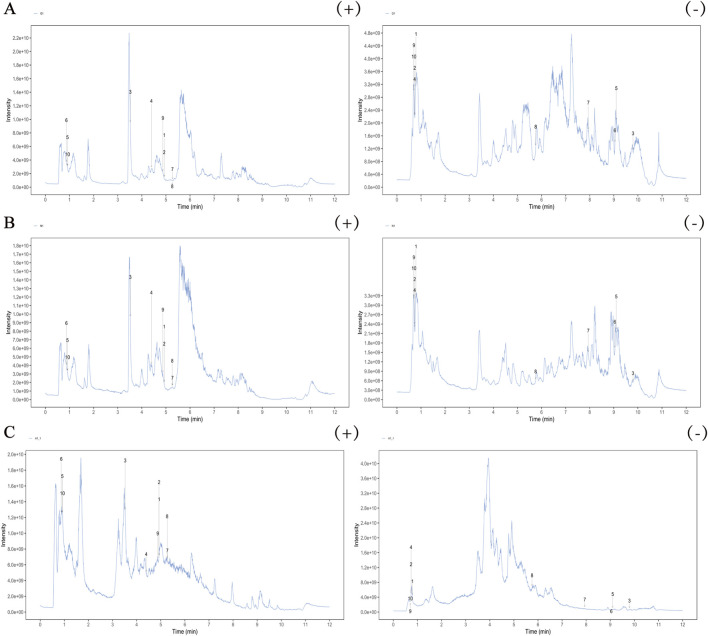
Total ion chromatogram of metabolites absorbed into serum after AVLE administration **(A)** rat serum after AVLE intervention, **(B)** blank serum, **(C)** AVLE).

UHPLC-OE-MS was used to perform positive and negative ion mode analysis on AVLE, rat serum after AVLE intervention, and blank serum. The results showed that 60 metabolites were identified as the metabolites absorbed into the serum of AVLE. Among them, 13 were prototype metabolites derived from AVLE, 17 were metabolites of the prototype metabolites and the remaining 30 were endogenous metabolites regulated by AVLE. The specific composition included 10 flavonoids, nine alkaloids, seven phenolic acids, etc (Figure 6; Supplementary Table S1).

### 3.7 Proteomics analysis

**FIGURE 7:**
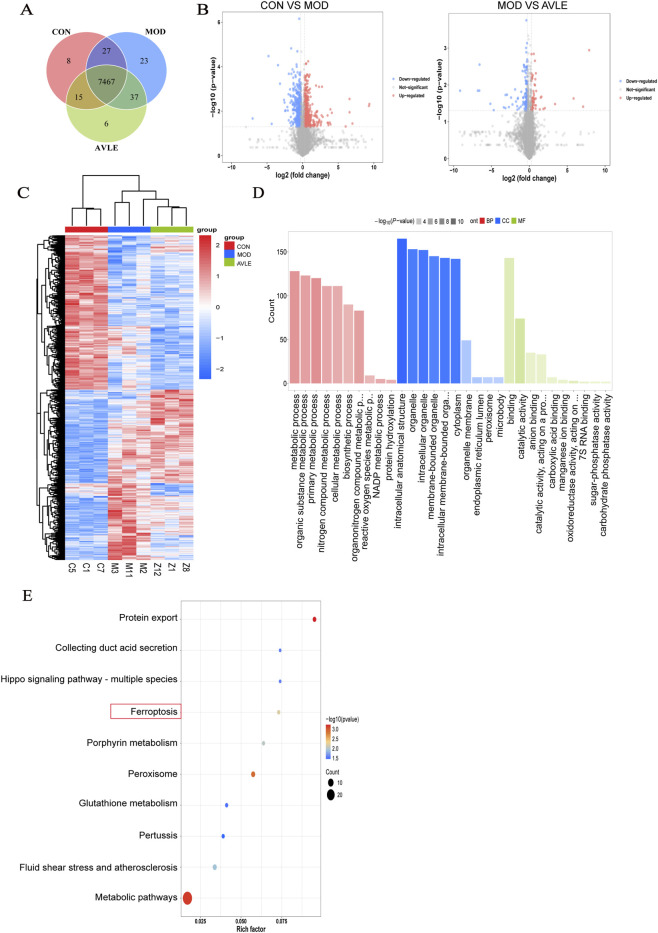
Proteomics analysis. **(A)** Venn diagram. **(B)** Volcano plot showing highly upregulated (red) and downregulated (green) proteins in comparisons between CON and MOD groups, and between MOD and AVLE groups. **(C)** Heatmap displaying differentially expressed proteins among CON, MOD, and AVLE groups. **(D)** GO enrichment analysis. **(E)** KEGG pathway enrichment analysis. n = 3.

A total of 7,583 proteins were detected in the present study. Venn diagram analysis illustrated that 7,467 proteins were co-expressed across all three experimental groups, which constituted the core protein set (Figure 7A). Relative to the CON group, 948 differentially expressed proteins (511 upregulated and 437 downregulated) were observed in the MOD group. Meanwhile, 189 differentially expressed proteins, including 67 upregulated and 122 downregulated ones, were identified between the AVLE group and MOD group (Figure 7B), and heatmap analysis verified remarkable differences between groups. Of the 189 proteins modulated by AVLE, 55 were abnormally expressed in the pathological state and notably restored after AVLE treatment (Figure 7C). GO analysis indicated that AVLE was closely associated with reactive oxygen species metabolism, oxidoreductase activity, and the function of intracellular membrane-bound organelles (Figure 7D). KEGG analysis demonstrated that these differential proteins were predominantly enriched in the ferroptosis pathway (Figure 7E). Notably, HO-1, a crucial regulator in iron metabolism and oxidative stress, was identified among the 55 proteins whose abnormal expression was reversed by AVLE. These findings suggest that the ferroptosis pathway may be involved in the renoprotective effects of AVLE.

### 3.8 Ferroptosis in the kidneys of db/db mice is suppressed by AVLE

**FIGURE 8:**
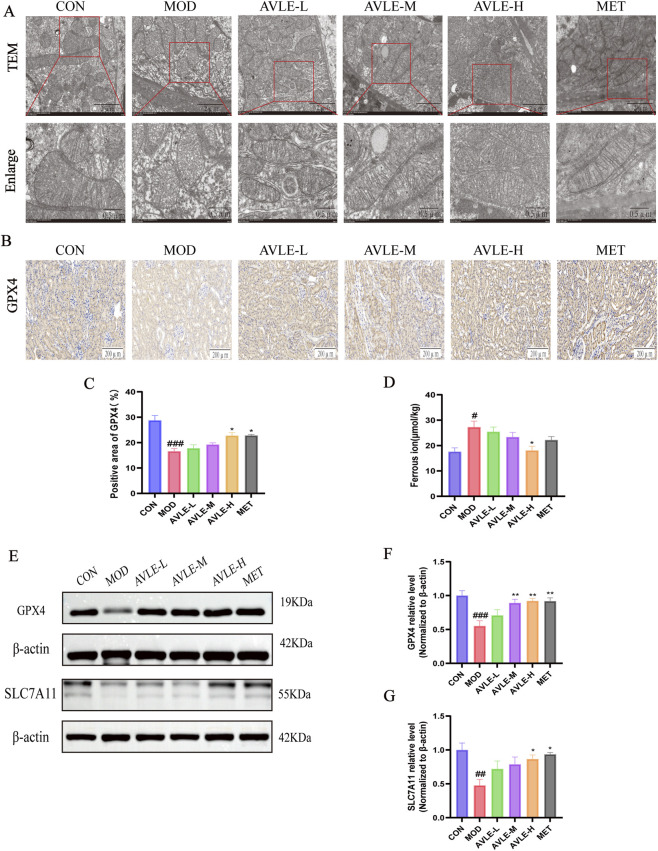
AVLE inhibits ferroptosis in db/db mice. **(A)** TEM result. Scale bar = 2 or 0.5 μm, n = 3. **(B,C)** Identification of GPX4 protein expression in kidney tissues by IHC. Scale bar = 200 μm, n = 3. **(D)** Ferrous iron levels in kidney tissues of each group, n = 6. **(E–G)** Western blot for GPX4 and SLC7A11 protein expression with semi-quantitative analysis. β-actin served as the internal control. All data are presented as mean ± SEM (n = 6). Compared with the CON group: ^#^ *p <* 0.05, ^##^ *p <* 0.01, ^###^ *p <* 0.001; Compared with the MOD group: *p < 0.05, **p < 0.01.

TEM images revealed distinctive ferroptosis-associated morphological alterations in renal tissue. The MOD group exhibited reduced or absent mitochondrial cristae, indicating ferroptosis in these mice. Treatment with AVLE and MET increased the number of mitochondrial cristae (Figure 8A).

IHC results revealed downregulation of GPX4 protein expression in the MOD group mice (*p* < 0.001), whereas both AVLE-H and MET groups exhibited markedly upregulated GPX4 expression (Figures 8B,C). The level of ferrous iron rose in the MOD group, while it declined in both the AVLE and MET groups (Figure 8D). Western blot results showed that the MOD group’s levels of GPX4 and SLC7A11 protein expression had decreased (*p* < 0.05). However, the administration of AVLE and MET groups countered these effects (Figures 8E–G).

### 3.9 Impact of AVLE on the expression of Nrf2 and HO-1 proteins in db/db mice

**FIGURE 9:**
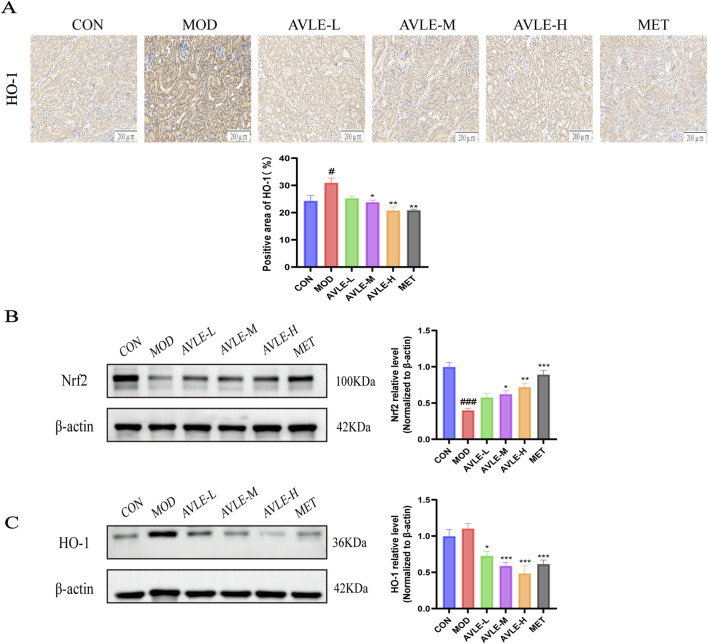
AVLE’s effects on the expression of the Nrf2 and HO-1 proteins in db/db mice. **(A)** IHC detection of HO-1 protein expression levels, n = 3. **(B,C)** Western blot investigation of Nrf2 and HO-1 protein expression levels. All data are expressed as mean ± SEM (n = 6). Compared with the CON group: ^#^ *p* < 0.05, ^###^ *p* < 0.001; Compared with the MOD group: ^*^ *p* < 0.05, ^**^ *p* < 0.01, ^***^ *p* < 0.001.

As identified by proteomic analysis, HO-1 expression was dysregulated in MOD mice and markedly restored following AVLE treatment. To further confirm the above speculation, IHC and Western blot were employed to detect the protein expression levels of HO-1 and its upstream regulator Nrf2. Immunohistochemical results revealed significantly elevated HO-1 protein expression levels in MOD mice, which decreased following AVLE intervention (Figure 9A). Western blot analysis revealed markedly reduced Nrf2 protein expression in MOD mice; AVLE therapy decreased HO-1 expression and increased Nrf2 protein (Figures 9B,C). The results demonstrated that AVLE can regulate the Nrf2/HO-1 signaling pathway.

### 3.10 Effects of AVLE on HG-induced renal injury and fibrosis-related proteins in HK-2 cells

**FIGURE 10:**
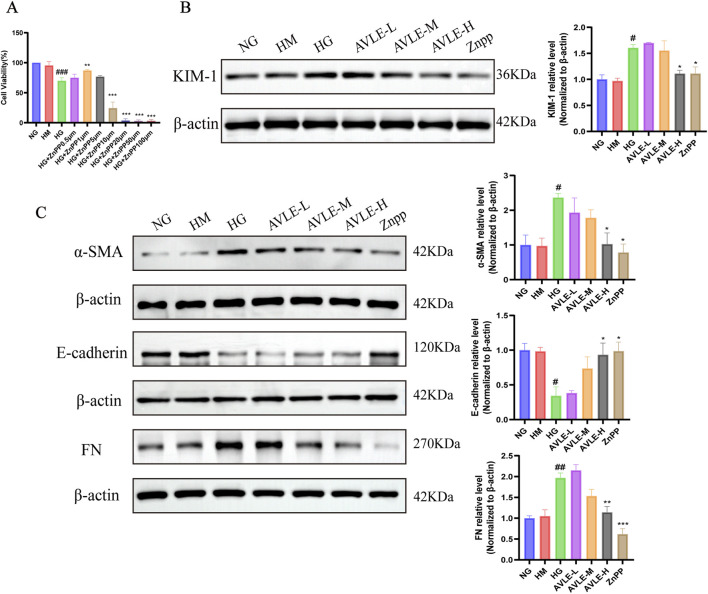
Effects of AVLE on HG-induced renal injury and fibrosis-related proteins in HK-2 cells. **(A)** ZnPP’s impact on the survival of HK-2 cells induced with high glucose. **(B,C)** Western blot study of each group’s expression levels of the proteins FN, E-cadherin, α-SMA, and KIM-1. All data are presented as mean ± SEM (n = 3). Compared with the NG group: ^#^ *p <* 0.05, ^##^ *p <* 0.01, ^###^ *p <* 0.001; compared with the HG group: ^*^ *p <* 0.05, ^**^ *p <* 0.01, ^***^ *p <* 0.001.

To explore the relationship between HO-1 expression levels and cell injury induced by HG, cells were treated with the HO-1 inhibitor ZnPP. The findings suggested that treatment with ZnPP improved cell viability, attaining its maximum concentration at 1 μM (Figure 10A). The expression of the KIM-1 protein was downregulated in the AVLE-H and ZnPP groups, whereas it was elevated in the HG (Figure 10B). E-cadherin expression was reduced in the HG, while α-SMA and FN protein expression levels were markedly increased. Both the AVLE-H and ZnPP groups induced a significant upregulation of E-cadherin, accompanied by a substantial decrease in α-SMA and FN levels. These results imply that ZnPP and AVLE protect HK-2 cells from fibrosis and damage brought on by high hyperglycemia (Figure 10C).

### 3.11 Ferroptosis in HK-2 cells triggered by HG is suppressed by AVLE

**FIGURE 11:**
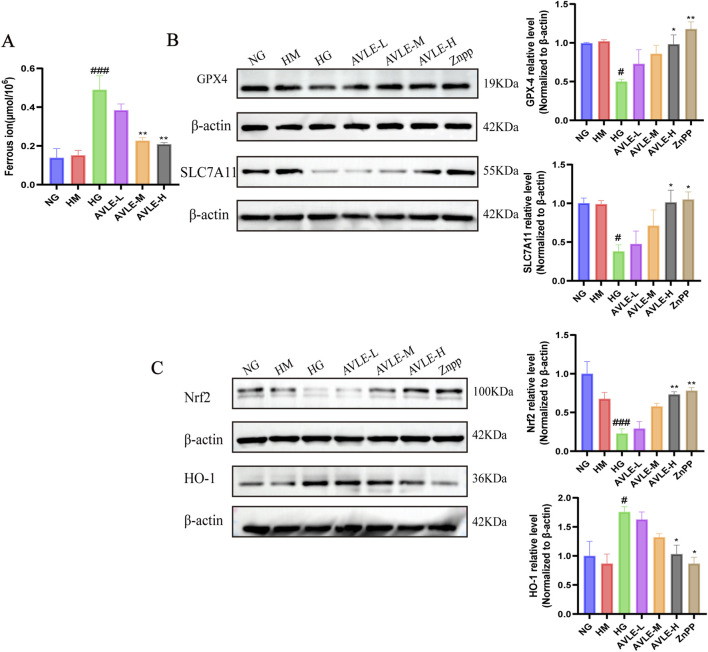
Impact of AVLE on Nrf2, HO-1 protein expression, and ferroptosis in HK-2 cells treated with HG. **(A)** Each group’s ferrous iron concentration was determined using a kit. **(B,C)** Each group’s levels of GPX4, SLC7A11, Nrf2, and HO-1 protein expression were identified by Western blot. All data are presented as mean ± SEM (n = 3). Compared with the NG group: ^#^ *p* < 0.05, ^##^ *p* < 0.01, ^###^ *p* < 0.001; compared with the HG group: ^*^ *p* < 0.05, ^**^ *p* < 0.01, ^***^ *p* < 0.001.

The HG group showed increased ferrous iron content, whereas the AVLE-M and AVLE-H groups exhibited reduced levels (Figure 11A). Western blot results indicated that GPX4 and SLC7A11 protein expression levels were downregulated in HG, but these changes were reversed in the AVLE-H and ZnPP groups (Figure 11B). According to these findings, AVLE has effects similar to those of ZnPP in inhibiting HG-induced ferroptosis in HK-2 cells.

### 3.12 Impact of AVLE on HK-2 cell Nrf2 and HO-1 protein expression under stimulation by HG

Consistent with *in vivo* experimental results, the Nrf2 protein expression was downregulated while HO-1 was upregulated in the HG group. AVLE and ZnPP groups exhibited upregulation of Nrf2 protein and downregulation of HO-1 protein expression. By contrast, the Nrf2 protein was significantly upregulated and the HO-1 protein expression was downregulated in the AVLE-H and ZnPP groups (Figure 11C). These findings demonstrate that AVLE modulates the Nrf2/HO-1 pathway.

## 4 Discussion

Between 20% and 40% of patients with diabetes are thought to have DKD (Gheith et al., 2015). Clinically, DKD treatment involves controlling the blood glucose, pressure, and lipids of patients. The management of DKD in a clinical setting focuses on regulating patients’ blood glucose levels, blood pressure, and lipid profiles. Renal protective effects in DKD can be achieved through the use of hypoglycemic agents, including MET and SGLT2 inhibitor (de Vos et al., 2018). In terms of blood pressure management, angiotensin receptor blockers or angiotensin-converting enzyme inhibitors may assist in preventing renal failure (Natale et al., 2024). However, when used alone or in combination with other medications, these drugs cause urinary tract infections and diabetic ketoacidosis (Mascolo et al., 2022), hyperkalemia, cough (Ku et al., 2024), and other side effects. Therefore, identifying appropriate pharmacological approaches has become urgently needed. *Apocynum venetum* L. extract can improve glucose-mediated protein damage (Yokozawa and Nakagawa, 2004). However, the potential mechanism of AVLE in DKD management remains incompletely understood.

Progressive decline in renal function is a core feature of DKD progression (Martín-Carro et al., 2023). In the present study, elevated levels of SCr and BUN, along with increased the expression of KIM-1, were observed in db/db mice; Cys-C levels also showed an increasing trend, collectively indicating impaired renal function. Concurrently, marked increases in 24 h-UP, mALB, and UACR were observed, indicating pronounced renal impairment and compromised glomerular filtration barrier function in the MOD group. AVLE treatment significantly reversed these changes, demonstrating its pharmacological effects in mitigating DKD-related renal decline and protecting the glomerular filtration barrier. Renal histopathology and ultrastructural analysis further confirmed AVLE’s renal protective effects. In MOD mice, typical DKD pathological changes were observed, including tubular epithelial cell edema, inflammatory infiltration, glycogen deposition, mesangial matrix expansion, and disorganized collagen fiber arrangement. Transmission electron microscopy further revealed severe podocyte damage, such as cytoplasmic dissolution, organelle swelling, and extensive podocyte foot process fusion. AVLE intervention significantly alleviated the aforementioned structural abnormalities, suggesting that it not only improves functional indicators but also delays the pathological progression of DKD.

Recent studies indicate that renal tubular lesions may appear in the early stages of DKD and are more closely associated with declining renal function than glomerular changes (Lu et al., 2025). Proximal tubular cells exhibit high sensitivity to hyperglycemia, making them a key therapeutic target (Mise et al., 2016; Hinden et al., 2022). Stimulating HK-2 cells with 60 mM hyperglycemia significantly reduced cell viability (Huang et al., 2022), whereas AVLE treatment increased cell viability in a concentration-dependent manner. LDH levels serve as a key indicator for assessing cellular injury (Zhang et al., 2023). AVLE intervention significantly reduced LDH release, confirming its cytoprotective effect.

Oxidative stress is one of the core factors driving DKD progression (Hughes et al., 2025). Chronic hyperglycemia induces mitochondrial dysfunction and activates NADPH oxidase, leading to excessive ROS production, which in turn triggers lipid peroxidation and depletion of the antioxidant defense system (Chae et al., 2023). In this study, AVLE treatment significantly elevated SOD and GSH levels in db/db mouse kidneys and in high-glucose-stimulated HK-2 cells, while reducing MDA content and intracellular ROS release, confirming its distinct antioxidant activity. Oxidative damage and inflammatory responses exhibit synergistic effects (Mo et al., 2025). Pro-inflammatory factors such as TNF-α and IL-6 are both induced by oxidative stress and can positively feedback to exacerbate ROS production. This study found that AVLE effectively suppressed the expression of these inflammatory factors both *in vivo* and *in vitro*. Persistent oxidation and inflammation ultimately drive renal fibrosis (Wang et al., 2025), a key pathological step in the progression of DKD to end-stage renal disease. AVLE intervention significantly downregulated the expression of pro-fibrotic factors α-SMA and FN while restoring the level of the epithelial marker E-cadherin. In summary, AVLE may exert multidimensional protective effects against DKD by synergistically enhancing antioxidant, anti-inflammatory, and anti-fibrotic capabilities.

Ferroptosis is an iron-dependent form of regulatory cell death driven by lipid peroxidation (Dixon et al., 2012). Iron accumulation catalyzes ROS production while simultaneously driving lipid peroxidation, ultimately leading to ferroptosis (Stockwell, 2022). Renal tubular epithelial cells exhibit particular sensitivity to this process under high-glucose conditions (Wang et al., 2024). Studies confirm that specific knockout of the key anti-ferroptotic gene GPX4 directly causes severe renal injury (Friedmann Angeli et al., 2014). These findings indicate that the kidney is a critical target for ferroptosis-mediated tissue damage. Typical ferroptosis-specific alterations were observed in db/db mice and high-glucose-stimulated HK-2 cells. Transmission electron microscopy revealed mitochondrial atrophy, reduced cristae, and even cristae disappearance in the MOD group mice. Concurrently, Fe^2+^ accumulation and downregulation of SLC7A11 and GPX4 were detected in both *in vivo* and *in vitro* experiments. Notably, AVLE treatment effectively reversed mitochondrial morphological alterations and ferroptosis-specific molecular abnormalities. These results indicate that the renal protective effects of AVLE are closely associated with direct inhibition of ferroptosis.

Nrf2 is a key regulator of antioxidant responses and modulates the activity of multiple ferroptosis-related proteins (Du et al., 2024). In this study, AVLE treatment restored Nrf2 expression, thereby enhancing overall antioxidant capacity, reducing ferrous iron levels, and inhibiting ferroptosis. As a downstream target of Nrf2, the balance between HO-1 products and free iron influences cellular oxidative stress and iron metabolism (Lv et al., 2025). In db/db mouse kidneys and high-glucose-stimulated HK-2 cells, HO-1 protein expression was upregulated, while AVLE significantly downregulated its expression. Inhibition of HO-1 using ZnPP effectively suppressed ferroptosis, with AVLE exhibiting comparable pharmacological effects to ZnPP. This indicates that AVLE inhibits ferroptosis in HK-2 cells by downregulating HO-1. Targeting HO-1 activity offers a novel pharmacological strategy for diabetic kidney disease (Liu et al., 2024).

Notably, HO-1 plays a dual role in ferroptosis regulation. Traditionally, HO-1 is regarded as a protective molecule: in DKD models, HO-1 expression is reduced, and ferroptosis inhibitors can upregulate its expression to mitigate damage to renal tubular epithelial cells (Li et al., 2025). However, recent studies suggest that HO-1 overexpression may exacerbate ferroptosis by releasing free iron and catalyzing ROS production (Chen et al., 2023), thereby intensifying iron overload and mitochondrial dysfunction (Luo et al., 2025). This controversy likely stems from HO-1’s regulation by multiple signaling pathways beyond Nrf2, with its net effect determined by the balance between antioxidant and pro-oxidant actions (Lv et al., 2025). Thus, the precise role of HO-1 in DKD warrants further investigation. This study demonstrates that AVLE inhibits ferroptosis by downregulating HO-1, suggesting a potential role for HO-1 regulation in AVLE’s mechanism.

This study primarily focuses on the overall pharmacological effects and mechanisms of action of AVLE in treating DKD; however, further validation using individual metabolites is still needed to confirm whether these components are responsible for AVLE’s nephroprotective effects. Second, AVLE exhibits selective action in the db/db mouse model, significantly reducing the extent of kidney damage and fibrosis, but without significantly modulating body weight or hyperglycemia levels. This suggests that AVLE may act through a mechanism distinct from traditional glucose-lowering pathways, potentially targeting renal pathological processes directly. Future studies combining AVLE with glucose-lowering drugs may help achieve more comprehensive therapeutic outcomes for patients with DKD.

## 5 Conclusion

**FIGURE 12:**
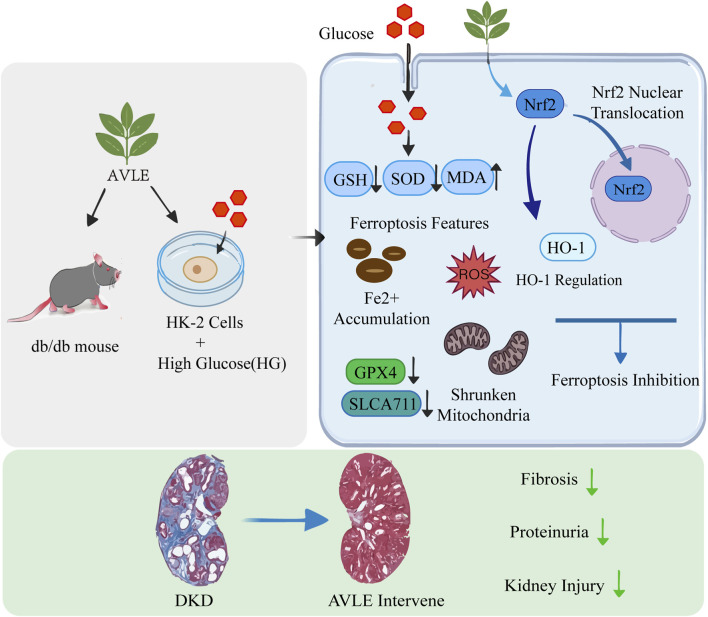
Schematic of the protective mechanisms of AVLE against diabetic kidney disease.

In summary, this study confirms that AVLE significantly improves renal function and mitigates histopathological damage. Its mechanism of action likely involves regulating the Nrf2/HO-1 signaling pathway, thereby inhibiting renal ferroptosis and fibrosis, providing new evidence for its pharmacological application in DKD (Figure 12).

## Data Availability

The data supporting the findings of this study are openly available in Figshare at 10.6084/m9.figshare.32012022.

## Supplementary material

SUPPLEMENTARY TABLE S1 Identification of metabolites absorbed into rat serum after AVLE administration detected by UHPLC-OE-MS.
